# The relationship between OPLL and metabolic disorders

**DOI:** 10.1038/s41413-025-00446-9

**Published:** 2025-10-24

**Authors:** Junfeng Wang, Ziheng Wei, Qingjie Kong, Yanqing Sun, Zhichao Zhang, Haiyuan Yang, Xiongsheng Chen

**Affiliations:** Department of Orthopedics, Shanghai General Hospital, Shanghai Jiao Tong University School of Medicine, Shanghai, China

**Keywords:** Bone, Metabolism

## Abstract

Ossification of the posterior longitudinal ligament (OPLL) is a degenerative disease characterized by progressive ectopic bone formation process, which can lead to severe neurological impairments and reduced quality of life. While the etiology of OPLL is generally considered multifactorial, there is no consensus regarding these contributing factors including genetic, endocrine, biomechanical, immune and lifestyle factors. Through accumulating evidence from multidisciplinary investigations, the pathophysiological connection between OPLL and endocrine-metabolic dysregulation is becoming increasingly clear. Nevertheless, comprehensive understanding of the relationship between the two is hindered by several problems, such as methodological limitations and inadequate mechanistic studies. This review takes a deep dive into the possible factors contributing to OPLL from all aspects of metabolism, including glucose metabolism, lipid metabolism, bone and mineral metabolism, leptin, vitamin, growth hormone/IGF-1 and sex hormones, highlighting their potential roles in the onset and progression of OPLL. Clarifying the etiology of OPLL and elucidating the underlying pathogenesis are crucial for advancing both early intervention strategies and therapeutic approaches in clinical management. Therefore, the endocrine and metabolic disorders in OPLL patients should become a focus of future research.

## Introduction

### Definition, epidemiology, and demography of OPLL

**Fig. 1:**
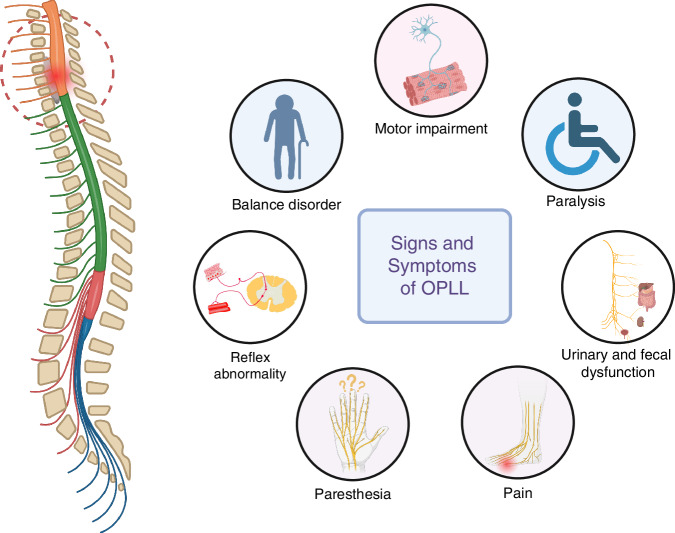
The spinal cord and nerve roots are compressed by the ossified mass originated from ligament tissue, resulting in various symptoms and signs according to the innervation

Ossification of the spinal ligament (OSL) represents a spectrum of pathological conditions marked by progressive heterotopic bone formation or calcium deposition in spinal connective tissues. This disease cluster encompasses three main subtypes: ossification of the posterior longitudinal ligament (clinically termed OPLL), ossification of the ligamentum flavum, and other ossified adjacent spinal support structures.^1^ Notably, OPLL manifests with heightened clinical severity due to the ligament’s unique anatomical positioning directly anterior to the spinal cord within the vertebral canal. The ossification process typically initiates at the posterior vertebral margins, leading to the formation of bone-like structures that progressively enlarge, potentially compressing neural elements. While OPLL may remain asymptomatic or manifest with nonspecific symptoms, even minor trauma can precipitate severe neurological deficits, including sensory and motor impairments, reflex abnormality, and urinary/fecal dysfunction. In extreme cases, paralysis may ensue, significantly impairing quality of life and imposing substantial socioeconomic burdens^1,2^ (Fig. 1).

**Fig. 2:**
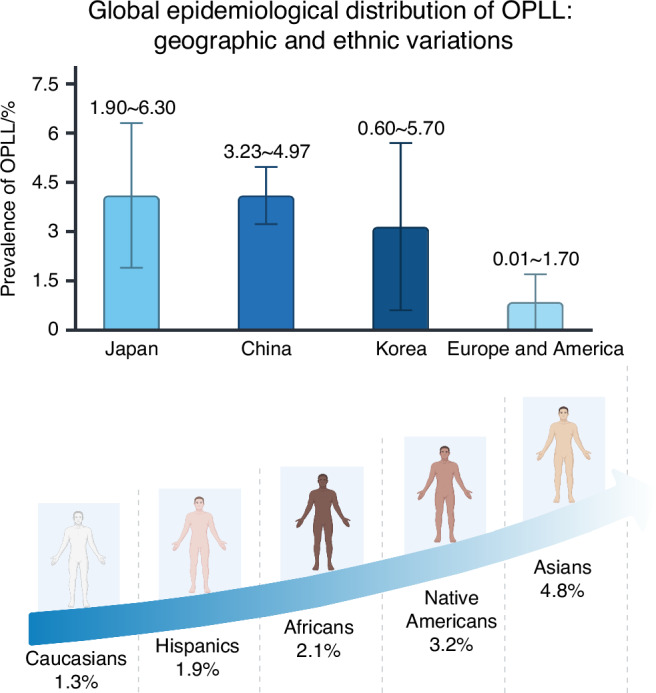
The left is the prevalence of C-OPLL across different regions. According to previous reports, the prevalence of C-OPLL was 1.90% to 6.30% in Japanese, 0.60% to 5.70% in Korean, 3.23% to 4.97% (95% confidence interval) in Chinese, and 0.01% to 1.7% in western populations. The right demonstrates the ethnic differences observed in the USA

OPLL predominantly affects the cervical spine, with the highest incidence observed at the C5 level, followed by C4 and C6. Thoracic and lumbar involvement is less common.^3,4^ It can sometimes coexist with other musculoskeletal disorders, such as ossification of the ligamentum flavum (OLF), diffuse idiopathic skeletal hyperostosis (DISH), and ankylosing spondylitis.^1,3,4^ Demographic studies have indicated that OPLL primarily occurs in individuals aged 40–60 years, with a higher prevalence in men (male-to-female ratio of 2:1). Symptomatic cases typically emerge in the fifth to sixth decades of life.^5,6^ The disease exhibits marked geographic and racial disparities, with higher prevalence rates observed in East Asian populations. For example, the prevalence of cervical OPLL (C-OPLL) has been documented as ranging from 1.9% to 6.3% in Japanese, 0.6% to 5.7% in Korean, with an average prevalence of 4.1% observed in Chinese populations.^7^ In contrast, prevalence rates in Western populations including the United States and Europe, are much lower, ranging from 0.01% to 1.7%.^8^ Notably, in a cross-sectional study comparing the prevalence of OPLL between different racial groups, Asian Americans also demonstrate the highest prevalence rates, underscoring the influence of genetic and ethnic factors^9^ (Fig. 2).

### Clinical presentation, diagnosis and management of OPLL

OPLL often progresses insidiously, with many patients remaining asymptomatic despite radiographic evidence of ossification.^2^ In its early stages, patients may experience mild discomfort, pain, or numbness in their hands, but as the disease advances and neural compression intensifies, symptoms worsen. Severe clinical manifestations may involve axial neck pain combined with neurocompressive phenomena such as radicular impairments and spinal cord dysfunction.^1^ Spine evaluations reveal that over 40% of patients will exhibit signs and/or symptoms of myelopathy.^10^ Key risk factors for neurological impairment include reduced spinal canal diameter (<6 mm), increased spinal mobility, lateral deviation of OPLL lesions, and spinal canal occupancy exceeding 50%–60% on cross-sectional imaging.^11–13^

Accurate diagnosis of OPLL relies on advanced imaging modalities, including radiography (X-rays), computed tomography (CT), magnetic resonance imaging (MRI), and three-dimensional (3D) reconstructions. Radiographic classification distinguishes OPLL into focal, segmental, continuous, or mixed types.^14^ The K-line, a radiographic parameter connecting the midpoints of the anteroposterior canal diameters at C2 and C7, aids in determining surgical approaches.^15^ CT imaging provides detailed visualization of ossified lesions, including their location, shape, size, and spinal canal occupancy, with severe cases often showing a characteristic “double-layer sign”.^16^ MRI is invaluable for assessing spinal cord compression, intramedullary signal changes, and predicting surgical outcomes.^12^

Management strategies for OPLL are tailored to disease severity and neurological status. Conservative treatments, such as cervical traction, bracing, nonsteroidal anti-inflammatory drugs (NSAIDs), activity modification, and physical therapy, are recommended for minimally symptomatic patients or those with high surgical risks.^17^ However, these measures do not mitigate the risk of future neurologic injury. Surgical intervention becomes necessary when patients exhibit advancing neurological deterioration or demonstrate persistent medullary compression refractory to comprehensive nonsurgical management.^18^ It aims to decompress neural structures, either by direct resection of ossified lesions or by expanding the spinal canal volume. There are two surgical approaches available: anterior options include discectomy with fusion and corpectomy, while posterior approaches encompass laminectomy with fusion (LF) or laminoplasty (LP). Complex cases may even require combined anterior-posterior decompression.^2,19^ Although surgical decompression remains the standard treatment for OPLL-induced neurological dysfunction, it carries significant risks of complications and does not halt disease progression, as evidenced by postoperative ossification progression in many cases.^20–22^

### Etiology, pathogenesis, and review objectives

OPLL primarily arises through endochondral ossification, a process involving several stages.^23,24^ Initially, fibrocartilaginous tissues undergo hypertrophy, driven by the proliferation of SOX9^+^ chondrocytes. This is followed by matrix mineralization, characterized by hydroxyapatite deposition, leading to the formation of calcified lesions. Hypertrophic chondrocytes within these lesions express RUNX2, a transcription factor essential for osteoblast differentiation. Ultimately, chondrocyte apoptosis, matrix degradation, and neovascularization result in the replacement of calcified cartilage with bone. As for etiology. OPLL is widely regarded as a multifactorial disorder,^1^ with genetic, endocrine, biomechanical, and lifestyle factors, as well as intracellular and extracellular cytokines all reported to influence its occurrence and progression.^25^ Despite extensive research, the precise mechanisms remain unclear, and the relative contributions of these factors are debated.

**Fig. 3:**
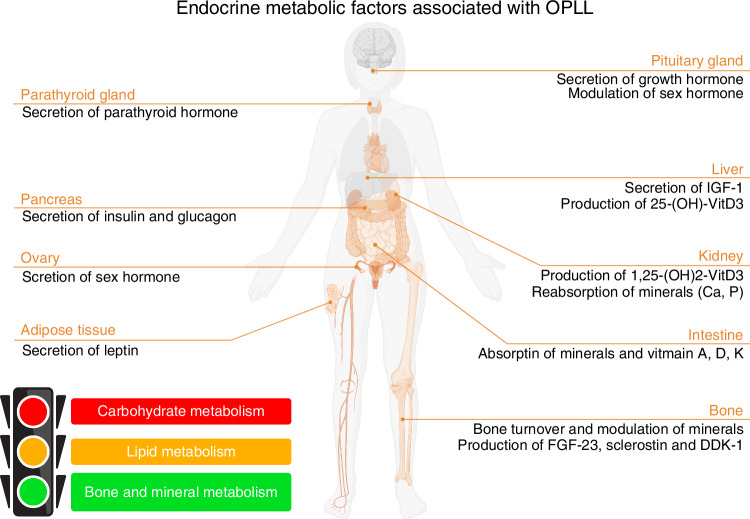
The human body diagram lists systemic endocrine-metabolic factors associated with OPLL

Emerging clinical evidence has revealed significant homeostatic imbalances in glucose metabolism (e.g., hyperglycemia with insulin resistance), lipid profiles (e.g., dyslipidemia), and bone remodeling processes (e.g., elevated bone mineral density) among patients with OPLL,^26–29^ establishing endocrine-metabolic dysregulations as a pathogenic cornerstone (Fig. 3). Nevertheless, current research remains predominantly observational—relying on cross-sectional clinical comparisons and epidemiological correlations, while mechanistic studies elucidating causal relationships between metabolic disorders and OPLL pathogenesis remain critically underexplored. Although recent investigations have identified several candidate biomarkers, such as hormones, calcium-phosphate metabolism markers, and bone turnover indicators,^26–29^ most of them primarily focus on comparative analyses between OPLL patients and healthy controls, without delving into underlying pathophysiological mechanisms. The inconsistent findings and insufficient mechanistic investigations severely hinder a deeper understanding of the relationship between OPLL and endocrine-metabolic factors.

Current therapeutic approaches, whether medical or surgical, fail to halt OPLL progression, underscoring the need for targeted therapies to arrest disease advancement. This review synthesizes and critically evaluates existing research on the interplay between OPLL and endocrine-metabolic dysregulations, offering a mechanistic understanding of disease pathogenesis through metabolic pathways. By identifying clinically actionable biomarkers for early diagnosis/intervention and druggable molecular targets, this work establishes a theoretical foundation for developing targeted therapeutic strategies centered on metabolic modulation, ultimately aiming to optimize OPLL management. Furthermore, as the first comprehensive reexamination of OPLL pathogenesis from an integrated metabolic perspective, this review not only clarifies existing knowledge gaps but also provides valuable insights for advancing future research in molecular diagnostics and personalized treatment approaches.

## Role of carbohydrate metabolism disorder

### High glucose

Diabetes mellitus, particularly type 2 diabetes mellitus (T2DM), represents one of the most prevalent disorders of carbohydrate metabolism. Epidemiological evidence suggests that T2DM is an independent risk factor for the development of OPLL.^30^ However, current research gaps persist in delineating the precise pathophysiological pathways linking diabetic dysregulation with ectopic spinal ossification. Hyperglycemia, a hallmark of diabetes, plays a pivotal role in diabetes-associated bone metabolism dysregulation. Experimental models demonstrate that elevated glucose levels potentiate reactive oxygen species (ROS) generation across diverse cellular populations, mediating critical processes including osteoblast differentiation,^31,32^ vascular calcification,^33^ and chondrocyte hypertrophy during endochondral ossification.^34^ Exposure to high glucose concentrations can induce alterations in gene expression, with varying effects on osteogenic differentiation depending on cell types. In vascular smooth muscle lineages, high glucose stimuli upregulate bone morphogenetic regulators (BMP-2/RUNX2), predisposing to pathological mineral deposition.^35^ In mesenchymal stem cells, glucose overload enhances both osteogenesis and chondrogenesis.^36,37^ Contrastingly, committed osteoprogenitor cells exhibit significantly attenuated differentiation capacity under high glucose conditions.^38,39^

Notably, differences in gene expression have been observed between posterior longitudinal ligament cells derived from OPLL patients and those from non-ossified ligaments. Cells from ossified ligaments exhibit osteoblast-like characteristics, including the expression of early osteogenic markers such as Runx2, alkaline phosphatase (ALP), and osteopontin,^40^ which is essential to obtain an osteoblast phenotype.^41^ Under hyperglycemic conditions (25 mmol/L glucose), research demonstrates enhanced BMP-2-mediated type I collagen production and upregulated expression of early osteogenic differentiation markers (ALP/RUNX2) in rodent spinal ligament fibroblasts. Mechanistically, the study elucidated three sequential pathological cascades: ROS overproduction initiating oxidative stress, subsequent activation of specific protein kinase C isoforms, and eventual suppression of the p38 MAPK signaling pathway. These sequential responses collectively promote abnormal collagen deposition and pre-osteoblastic genetic programming in these specialized cells.^42^ While the gluco-ossification correlation remains contentious in certain cohorts,^29^ chronic hyperglycemia appears to be a plausible etiologic contributor to T2DM-OPLL comorbidity.

### Hyperinsulinemia

Extensive population-based research has identified obesity (BMI ≥ 25 kg/m²), impaired glucose tolerance, and non-insulin-dependent diabetes mellitus (NIDDM) as significant risk factors for OPLL.^30,43,44^ These metabolic disturbances frequently coincide with disrupted insulin signaling pathways and elevated serum insulin levels. This pathophysiological correlation has prompted investigators to explore insulin’s regulatory functions in ectopic bone formation processes. Recently, quantitative analyses have demonstrated that dynamic insulin secretion patterns directly correlate with radiographic progression of spinal ossification, with postprandial insulin production metrics showing particular promise as prognostic biomarkers of OPLL.^45^ In addition to OPLL patients, hyperinsulinemia is also observed in animal models of OPLL, such as leptin receptor-deficient Zucker rats which demonstrate characteristic hypersecretion of pancreatic hormones.^46^

These observations in OPLL people and animal models align with cellular-level findings, where supraphysiological insulin concentrations (≥100 nmol/L) are shown to significantly potentiate ectopic mineralization processes in vascular lineage cells.^47^ Strikingly, mechanistic studies have revealed significant overlap between two key biological pathways: the insulin-responsive PI3K/Akt pathway and BMP-2-activated osteogenic signaling networks. This convergence suggests common molecular mechanisms may simultaneously regulate metabolic processes and bone formation.^48,49^ Spinal ligament cells possess insulin receptor substrate-1 (IRS-1), enabling them to detect circulating insulin and make a response. Research findings demonstrate that chronically elevated insulin levels direct cellular differentiation processes within spinal ligament cells through a metabolic regulatory system. Specifically, through insulin receptor-triggered PI3K-Akt pathway activation, coupled with the inhibition of ERK-mediated growth regulation, hyperinsulinemia creates conditions favoring both cell proliferation and BMP-2-induced osteogenic differentiation.^50^ This mechanistic insight provides a potential explanation for how prolonged hyperinsulinemia may drive OPLL progression in individuals with type 2 diabetes.

### Advanced glycation end products (AGEs)

Advanced glycation end products (AGEs) represent a class of pathologically modified biomolecules generated through non-enzymatic glycation reactions, involving covalent crosslinking between reducing sugars and macromolecular substrates like proteins, lipids, and nucleic acids. Their systemic accumulation increases with the course of natural aging and diabetes, and exhibits a strong correlation with diabetes severity.^51^ High AGE concentrations have been linked to aberrant mineralization, ectopic calcification, and impaired osteoblast differentiation.^52,53^ And emerging metabolomic evidence has demonstrated that circulating pentosidine concentrations, a cardinal AGE biomarker, are quantitatively associated with OPLL presence.^54^ Histopathological characterization of ossified spinal ligaments confirms the presence of AGEs and their receptors (RAGEs). Crucially, in vitro exposure to AGEs (1 mg/mL) greatly upregulates the expression of osteogenic inducers such as BMP-2 and Cbfa1 in ligament cells from OPLL patients.^55^ These findings position the AGE-RAGE axis as a critical mediator connecting metabolic dysregulation to pathological ectopic ossification. Clinically, the diagnostic potential of AGEs has prompted adoption of non-invasive detection techniques like skin autofluorescence measurement. Research data reveal individuals with thoracic OPLL exhibit significantly higher AGE scores compared to those without OPLL or with cervical OPLL, underscoring the clinical value of AGE quantification in differential diagnosis of spinal ligament ossification subtypes.^56^

Overall, the triad of chronic hyperglycemia, compensatory hyperinsulinemia, and advanced glycation end-product deposition, pathognomonic features of type 2 diabetes mellitus, constitute pathophysiological convergence points in both diabetic metabolic dysregulation and OPLL development. Although these metabolic disturbances provide a framework for understanding the link between OPLL and diabetes, comprehensive mechanistic investigations remain imperative to decipher their spatiotemporal interplay and identify therapeutic targets for comorbid disease modulation.

## Role of lipid metabolism disorder

Lipid metabolism has emerged as a novel and promising area of research in the context of OPLL. Epidemiological analyses document that OPLL patients demonstrate significantly higher prevalence rates of dyslipidemia compared to non-OPLL controls, characterized by elevated serum levels of total cholesterol and triglyceride,^57,58^ underscoring the potential role of lipid metabolism dysregulation in OPLL pathogenesis. For instance, Fukuda et al. identified dyslipidemia as a novel risk factor for OPLL based on serum lipid profiles, suggesting visceral fat obesity and increased focal mechanical stress due to excess weight may contribute to the condition.^58^ Zhang et al. utilized Mendelian randomization analysis to reveal that elevated total cholesterol and low-density lipoprotein cholesterol (LDL-C) levels could increase susceptibility to OPLL.^59^ Additionally, Endo et al. highlighted non-alcoholic fatty liver disease (NAFLD) as a potential risk factor for OPLL progression, with findings showing a higher prevalence of NAFLD in middle-aged OPLL patients and a negative correlation between liver-to-spleen ratio and ossification severity.^60^

The pathophysiological interplay between lipid dysregulation and ectopic calcification has garnered significant attention. Abnormal dyslipidemia has been linked to the development of vascular calcification. Specifically, through the oxidative damage-mediated apoptosis of vascular smooth muscle cells and downregulation of endogenous calcification inhibitors, dyslipidemia-induced oxidative stress and sustained inflammatory signaling create a permissive microenvironment for cellular phenotype transformation, ultimately driving osteoblastic transdifferentiation and subsequent matrix mineralization.^61^ Interestingly, these cellular events exhibit striking similarities with the conditions observed in spinal ligament calcification. Central to these mineralization processes is the Wnt/β-catenin signaling pathway, which has been identified as a shared regulatory axis in both vascular calcification and bone formation.^62,63^ Among numerous signaling molecules in Wnt network, LRP5, a transmembrane co-receptor integral to Wnt signal transduction cascades, functions as a molecular nexus connecting lipid metabolism with skeletal homeostasis.^64^ In osseous tissues, LRP5 associates with Wnt ligands and Frizzled receptors to form a ternary signaling complex. This molecular assembly initiates a downstream cascade culminating in β-catenin accumulation and nuclear translocation, thereby resulting in the transcription of osteogenic master regulators such as Cbfa-1.^65,66^ Studies have shown mild dyslipidemia can trigger canonical Wnt/β-catenin pathway via LRP5.^67^ Furthermore, pro-oxidative conditions induced by hyper-LDLemia are mechanistically linked to vascular calcification processes via dual LRP5/LRP6-dependent Wnt pathway activation.^66,68^ These discoveries collectively suggest a potential mechanism by which dysregulated lipid profiles, particularly elevated LDL concentrations, may predispose to spinal ligament ossification through Wnt signaling pathway-mediated disruption of bone homeostasis. While these findings provide compelling mechanistic insights, direct experimental evidence linking lipid-mediated Wnt signaling dysregulation to OPLL pathogenesis remains limited. Further investigations are warranted to delineate the exact role of Wnt signaling in lipid metabolism-associated ectopic ossification of the posterior longitudinal ligament.

Metabolomic profiling studies have provided additional insights into the role of fatty acid metabolism in OPLL. Tsuji et al. illuminated distinct fatty acid (FA) profiles with significant elevation of fatty acid-carnitine conjugates in OPLL patients, reinforcing the involvement of lipid metabolism dysregulation in the pathophysiology of this disease.^69^ Previous investigations have reported that fatty acid derivatives can modulate bone remodeling processes through dual receptor-mediated pathways, including the activation of free fatty acid receptor 4 (FFAR4) signaling cascades and potentiation of parathyroid hormone type 1 receptor (PTH1R) activity. These interactions create a pro-osteogenic microenvironment by enhancing osteoblastgenesis while suppressing osteoclastogenesis.^69^ Therefore, these elevated lipid intermediates may function as molecular mediators, thus promoting ligamentous ossification processes in OPLL patients. Mead acid and n-3 polyunsaturated fatty acids (PUFAs) are also hypothesized to play a role in OPLL pathogenesis, but comparative lipidomic analyses failed to demonstrate significant concentration differences between OPLL cohorts and control groups.^70^ Conversely, quantitative profiling uncovers marked elevation of palmitic acid levels in OPLL patients, with a positive correlation with OPLL risk. However, the reason is unclear and their true relationship remains doubtful.^70^ The precise biological significance of specific fatty acids in OPLL pathogenesis still requires further research.

## Role of mineral and bone disorder

### Minerals

The relationship between mineral metabolism disorders and spinal ligament ossification was first reported by Adams and Davis.^71^ Calcium and phosphorus represent essential mineral components crucial for bone health. In normal physiological states, the balance of these minerals is predominantly maintained by calcium-modulating hormones, which are vital for proper bone homeostasis. However, clinical observations have identified disrupted mineral metabolism in OPLL patients, especially those with coexisting endocrine disorders affecting mineral modulation, such as hypoparathyroidism and vitamin D-resistant rickets,^71–74^ suggesting a potential role of aberrant calcium and phosphate metabolism in this pathogenesis.

Although the co-occurrence of Vitamin D-resistant hypophosphatemic rickets and OPLL has been well documented,^75^ the accurate incidence of OPLL in patients with this rickets variant remains uncertain due to the limited samples. Vitamin D is well known for its indispensable role in maintaining the mineral and bone homeostasis. While serum measurements show comparable levels of vitamin D metabolites (25-OHD and 1,25-(OH)2D) and circulating calcium between OPLL patients and healthy individuals,^27,28,76^ functional evaluations reveal distinct abnormalities.^77^ Specifically, certain OPLL patients demonstrate markedly diminished calciuric responses following oral calcium administration. Furthermore, subsequent investigations disclose a strong association between this hypocalciuric response and the advancement of OPLL.^78^ The urinary calcium response to oral calcium intake indeed serves as an indicator of intestinal calcium absorption efficiency, which is primarily regulated by active vitamin D metabolites. These observations have led researchers to hypothesize that localized vitamin D activity impairment or tissue-level active vitamin D deficiency, rather than systemic alterations, may underlie OPLL pathogenesis.^8^ Comparative analysis of bone mineral density (BMD) between OPLL patients and individuals with vitamin D-resistant rickets provides additional validation for this hypothesis. In vitamin D-resistant rickets, bone metabolism abnormalities manifest as decreased BMD and increased unmineralized bone matrix. In contrast, OPLL patients typically exhibit elevated spinal and even systemic BMD regardless of their gender.^79,80^ BMD, an important marker of mineralization status, is known to be influenced by active vitamin D’s ability to regulate both bone-forming and bone-resorbing activities.^29^ However, the precise cellular and molecular mechanisms through which active vitamin D enhances mineralization while suppressing bone resorption in OPLL remain unclear, highlighting the need for further investigation into this complex metabolic pathway.

In addition to vitamin D-resistant rickets, a notable prevalence of OPLL among patients with hypoparathyroidism has also been identified, though the exact nature of this association remains a matter of debate.^73^ The biological basis for this potential connection may lie in the physiological functions of parathyroid hormone (PTH), which regulates calcium and phosphate metabolism by increasing serum calcium while decreasing phosphorus levels. In fact, PTH’s role in bone metabolism is particularly complex, exhibiting both catabolic and anabolic effects depending on its temporal pattern and duration of exposure.^81^ At the cellular level, research has revealed distinct responses to PTH between ossified and normal ligament tissues. Ligament cells from OPLL patients demonstrate osteoblast-like characteristics when exposed to PTH, contrasting with non-ossified ligament cells from healthy individuals, which show no such osteoblast properties.^40^ This differential response suggests that ossified ligament cells possess a unique capacity to react to PTH in a manner associated with bone formation. Recent comparative studies have added another layer of complexity by examining OPLL patients with and without DISH. These investigations found significantly higher BMD and intact PTH (i-PTH) levels in OPLL patients with DISH compared to those without DISH.^82^ However, the specific relationship between DISH and PTH levels remains unexplored, and the precise role of PTH in ligament ossification continues to be poorly understood.

Fibroblast growth factor 23 (FGF-23), a phosphotropic hormone primarily secreted by osteocytes and osteoblasts, plays a critical role in phosphate regulation through the FGF23/Klotho signaling pathway. This cytokine exerts its effects by suppressing active vitamin D synthesis, reducing intestinal phosphate absorption, and inhibiting renal phosphate reabsorption, thereby maintaining phosphate homeostasis.^83,84^ Elevated circulating FGF-23 levels and associated hypophosphatemia have been consistently observed in both OPLL animal models (Enpp1 ttw/ttw mice) and human patients,^27,28,85^ suggesting a potential involvement of FGF-23-regulated phosphate metabolism in OPLL pathogenesis. Moreover, recent clinical studies even propose high levels of FGF-23 and hypophosphatemia as potential diagnostic markers for OPLL.^83^ The connection between phosphate metabolism disorders and OPLL is further supported by the frequent co-occurrence of rickets in OPLL patients.^75,86^ Similar to OPLL, rickets is characterized by reduced serum phosphate levels, indicating that abnormal phosphate metabolism may be a common feature of both conditions. However, this hypothesis remains controversial, as some studies have found no significant differences in serum phosphate levels between OPLL patients and healthy controls, despite elevated FGF-23 concentrations.^27^ The potential mechanisms linking FGF-23 to OPLL development remain obscure. Experimental evidence suggests that FGF-23 may inhibit chondrogenesis and accelerate the transition of chondrocytes from proliferation to hypertrophy, potentially contributing to endochondral ossification processes observed in OPLL.^87^ FGF-23-phosphate metabolism axis may as a promising area for future research into OPLL pathogenesis and potential therapeutic targets.

While significant advances have been made in identifying calcium-phosphate disturbances as potential contributors, the mechanisms linking mineral metabolism abnormalities to the development of OPLL remain incompletely understood. The rare co-occurrence of OPLL with endocrine disorders such as vitamin D-resistant rickets and hypoparathyroidism offers valuable insights into calcium-phosphate dysregulation mechanisms. However, the scarcity of well-documented cases and absence of comprehensive case-control studies have hindered systematic investigation of these metabolic associations. Beyond calcium and phosphorus, recent epidemiological data have expanded the investigative scope to fluoride, another essential mineral influencing bone metabolism and mineralization.^88^ A significant correlation between fluorosis and OPLL incidence has been established, with increased disease severity being associated with elevated urinary fluoride levels,^89^ suggesting that fluoride’s osteogenic properties may contribute to pathological bone formation in spinal ligaments.

### Bone turnover markers

OPLL patients typically exhibit increased bone mineral density,^80,90^ suggesting enhanced bone formation activities linked to this disease. Research has explored connections between OPLL and various biochemical indicators of bone remodeling, such as type I collagen derivatives (PICP, ICTP, PINP), bone-specific proteins (osteocalcin, osteopontin), urinary collagen breakdown products (Pyr, Dpyr), and tartrate-resistant acid phosphatase isoform5b (TRAP5b).^27,74,76,91–93^ Nevertheless, due to limitations, such as a limited sample size, lack of comparison groups, and insufficient control for variables influencing bone metabolism involving age, gender, BMI, and renal function, these investigations have yielded conflicting outcomes, failing to establish definitive conclusions. Consequently, well-designed studies with standardized protocols and adequate sample sizes are essential for clarification. Despite controversy, it remains evident that abnormal bone turnover marker profiles are associated with OPLL’s pathological mechanisms.

### Wnt signaling markers

It is well known that Wnt signaling cascade represents a crucial regulatory system in human physiology. As an osteogenic pathway, the Wnt/β-catenin pathway serves as a key modulator of bone formation through promoting the propagation of osteoprogenitor cells and inhibiting the programmed cell death of osteoblasts.^94–96^ Among various regulators of this pathway, sclerostin and dickkopf-1 (DKK-1) have emerged as the most extensively studied endogenous inhibitors. Sclerostin, a glycoprotein secreted by osteocytes, exerts its inhibitory effect by interacting with low-density lipoprotein receptor-related proteins (LRPs), thereby blocking Wnt ligand binding.^97^ DKK-1 also acts as an antagonist of Wnt signaling and similarly regulates the pathway through its binding to LRP5/6 co-receptors, though its mode of action differs from sclerostin.^98^ In the context of posterior longitudinal ligament ossification, alterations in these Wnt inhibitors have been widely observed. Most studies reported elevated serum sclerostin levels alongside reduced DKK-1 concentrations in OPLL patients, while some investigations found no significant changes in DKK-1 levels.^27,74,92,99^ Another two studies also investigated the relationship between DKK-1 and DISH, but their findings were conflicting.^100,101^

Notably, increased sclerostin production in older individuals with OPLL has been linked to both higher bone mass and reduced bone turnover, with decreased DKK-1 levels potentially representing a compensatory response to elevated sclerostin.^74^ Therefore, the discrepancies in these studies may reflect the complex interplay between Wnt signaling components and systemic factors such as age and bone mass. At the cellular level, research have demonstrated that OPLL ligament cells exhibit reduced DKK-1 expression compared to normal controls. Further explorations reveal this downregulation correlates with enhanced Wnt/β-catenin pathway activity, as indicated by increased β-catenin stability and transcriptional activation. Additionally, DKK-1 can inhibit BMP-2-induced osteogenic differentiation in ligament cells through its suppression of Wnt signaling.^99^ These findings collectively position DKK-1 as a critical negative regulator of ligament ossification. Targeting the Wnt pathway through DKK-1 modulation may offer a promising therapeutic approach for OPLL management.

The canonical Wnt signaling pathway can also influence bone remodeling through boosting osteoprotegerin (OPG) production while suppressing receptor activator of nuclear factor κB ligand (RANKL) expression in osteoblasts, thereby creating an anti-resorptive environment.^102^ As a soluble receptor secreted by osteoblasts, osteoprotegerin plays a pivotal role in bone mass regulation by competitively binding to RANKL and preventing its interaction with RANKL receptors, thus inhibiting osteoclast formation and activity.^103^ Comparative analyses have demonstrated significantly elevated concentrations of both osteopontin and OPG in ligament tissues from OPLL patients compared to non-affected individuals. Conspicuously, tissue OPG levels were found to be sixfold higher than corresponding serum concentrations, suggesting a localized mechanism of bone formation regulation in OPLL.^104^ However, this tissue-specific upregulation contrasts with systemic measurements, as different studies show no significant difference of serum OPG levels between OPLL and non-OPLL groups.^92^ Further investigations are needed to elucidate the distinct patterns of molecular expression between local tissue environments and systemic circulation in OPLL pathogenesis.

To date, current understanding of bone metabolism dynamics in OPLL remains incomplete, characterized by conflicting research findings and unresolved questions. A fundamental uncertainty persists regarding the causal relationship between abnormal bone metabolism and OPLL development - whether it serves as a primary driver or secondary consequence of the disease. Addressing these questions demands comprehensive clinical studies combined with deep mechanistic investigations at cellular and molecular levels. Most importantly, advancing our knowledge of bone metabolism in OPLL holds significant clinical potential. A thorough understanding could enable the development of predictive biomarker systems, allowing for early identification of at-risk individuals and monitoring of disease progression. Such biomarkers could revolutionize clinical management by facilitating timely interventions and personalized treatment strategies. As discussed above, the Wnt signaling pathway emerges as a promising target for drug development. Identification of novel regulatory components within this pathway could lead to innovative pharmacological approaches to restore bone homeostasis, potentially offering more effective targeted treatment options than current symptom-focused management strategies.

## Other endocrine-metabolic factors

### Vitamin

In addition to aforementioned vitamin D, vitamins associated with OPLL pathogenesis also involve vitamin A and vitamin K in spite of conflicting evidence surrounding their roles in this disease. Early clinical observations associated excessive vitamin A intake with heterotopic ossification phenotypes including OPLL and DISH, particularly in patients receiving long-term vitamin A supplementation, suggesting a potential causal relationship.^105,106^ Preclinical models further corroborated these findings, demonstrating that sustained vitamin A administration induces osteophyte formation and heterotopic ossification in tendons and joint capsules within six-month exposure windows.^107^ Additionally, biochemical analyses have identified elevated circulating retinol and retinol-binding protein (RBP) concentrations in OPLL patients, with pronounced elevations observed in females.^108^ However, contemporary epidemiological data present contradictory evidence, revealing reduced dietary vitamin A intake and correspondingly lower serum levels in early-onset OPLL cases. Furthermore, quantitative assessments disclose a negative correlation between serum vitamin A and OPLL severity, challenging previous assumptions about vitamin A’s role in disease progression.^109^ Retinoic acid signaling is regarded as a potent inhibitor of chondrogenesis,^110^ and nuclear retinoic acid receptor agonists have been proved to inhibit heterotopic ossification by suppressing endochondral ossification.^111^ Based on these findings, vitamin A insufficiency is conjectured to facilitate OPLL progression by diminishing endogenous inhibitory effects on endochondral ossification.^109^ While accumulating evidence has implicated abnormal vitamin A metabolism in OPLL pathophysiology, the role of vitamin A remains obscure. Further exploration and mechanistic studies are warranted to delineate the specific association.

Vitamin K functions as an essential enzymatic cofactor for γ-glutamyl carboxylase, catalyzing the post-translational modification of substrate proteins through the conversion of glutamate residues into γ-carboxyglutamate moieties. This biochemical transformation is critical for the function of vitamin K-dependent proteins (VKDPs) that regulate hemostasis, vascular calcification, and bone metabolism.^112^ Clinical investigations have identified prolonged clotting times and reduced plasma protein C levels in patients with OPLL, indicating potential vitamin K deficiency in OPLL pathogenesis.^24^ Besides, experimental studies utilizing ttw mice, a well-characterized cervical OPLL model, demonstrate that dietary vitamin K supplementation significantly attenuates cervical ligament calcification and improves motor function, suggesting vitamin K’s protective effects against ectopic mineralization and its potential as a therapeutic strategy for OPLL.^24^ However, contrasting evidence emerges from epidemiological analyses, with a recent case-control investigation reporting much higher serum menaquinone-4 (MK-4, a form of vitamin K2) levels in male OPLL patients compared to matched controls.^113^ While some experimental data confirm vitamin K2’s dual regulatory effects on bone metabolism—enhancing osteoblast function while suppressing osteoclast activity, in vitro assays using OPLL-derived ligament cells demonstrated no significant MK-4-induced ALP elevation or osteoblastic activation.^113^ These paradoxical findings highlight the need for mechanistic investigations into vitamin K’s tissue-specific effects and its metabolic interplay with VKDPs in ectopic ossification processes.

### GH/IGF-1

Clinical observations have documented co-occurrence of spinal ligament ossification disorders with acromegaly, which is characterized by excessive circulating growth hormone (GH) and insulin-like growth factor 1 (IGF-1).^114–116^ The GH/IGF-1 paracrine signaling axis is well established as a critical regulator of bone formation and remodeling processes. An in vitro investigation has revealed pronounced IGF-1 expression patterns in ossified posterior longitudinal ligament tissues compared to non-pathological tissues from normal individuals.^117^ When exposed to IGF-1, the upregulation of DNA replication and type I collagen precursor synthesis were observed in both OPLL and non-OPLL cell lines. Crucially, significantly enhanced ALP induction was identified in OPLL-derived cells following IGF-1 stimulation,^117^ indicating that IGF-1 signaling preferentially activates osteogenic programming in these ossification-prone ligament cells, potentially contributing to localized ectopic mineralization characteristic of OPLL pathology. However, contemporary analyses show no statistical differences in circulating GH/IGF-1 concentrations between acromegaly patients exhibiting spinal ligament ossification and those without, nor a significant correlation between ossification index and GH or IGF-1 levels. Intriguingly, elevated serum growth hormone receptor (GHBP) levels are detected in OPLL patients.^116^ While the anabolic effects of GH signaling in skeletal development are well elucidated, its contributions to spinal ligament ossification processes remain poorly defined, necessitating further mechanistic investigations into this endocrine-osseous axis.

### Leptin

Obesity has been widely recognized as a significant contributor to the development of OPLL.^118–120^ The metabolic state of obesity is frequently accompanied by elevated circulating leptin, a hormone encoded by the obesity gene and predominantly secreted by fat cells. This hormone plays a pivotal role in translating adipose tissue signals into bone metabolic responses, with multifaceted mechanisms involving both central and peripheral pathways towards bone remodeling. Centrally, leptin exerts inhibitory effects on osteogenesis and bone resorption through hypothalamic-sympathetic neural pathways, while peripherally, it stimulates bone formation by enhancing the multiplication and specialization of bone marrow mesenchymal cells into osteoblasts.^121^ Numerous studies have linked leptin to OPLL pathogenesis, with abnormally high leptin levels detected in both genetically obese Zucker rat models and female OPLL patients.^122,123^ Research by Ikeda and colleagues establishes a direct relationship between circulating leptin concentrations and the spinal regions affected by OPLL. Furthermore, these increased leptin levels show a positive association with insulin concentrations, indicating that the combined effects of elevated leptin and insulin may drive pathological ossification processes in spinal ligaments.^122^ Another study by Takahata’s group reveals that patients with extensive OPLL involvement present with greater body mass indices (BMI), altered adipokine ratios, and elevated osteocalcin levels compared to those with single-region OPLL. And adiponectin-to-leptin ratio negatively correlated with OPLL severity is proposed as a potential marker for adipokine dysregulation that may underlie widespread spinal ligament ossification in obese populations.^121^

Leptin’s involvement in spinal ligament ossification processes has been explored a lot in these years. Previous experimental evidence demonstrates leptin’s capacity to promote cell proliferation and osteogenic differentiation in various cell types, including embryonic cells, bone marrow stromal cells, and osteoblasts.^124–127^ A separate investigation by Fan’s team discloses that leptin specifically upregulates bone-specific gene markers (ALP and OCN) in ligamentum flavum cells from ossification patients, with no similar effects observed in normal ligament cells. These osteogenic responses exhibit concentration- and time-dependent patterns, though only at supraphysiological doses.^124^ Further mechanistic studies by Feng et al. show that leptin treatment in ossified ligament cells from cervical OPLL samples not only enhances osteogenic marker (ALP and OCN) expression and mineralization capacity but also activates multiple signaling cascades including ERK1/2, p38 MAPK, and JNK pathways.^128^ Chen’s group provides additional insights by examining the combined effects of leptin signaling and mechanical stress on posterior longitudinal ligament cells, demonstrating their synergistic promotion of osteogenic differentiation through MAPK, JAK2-STAT3, and PI3K/Akt pathways. In their studies, distinct expression patterns of relevant molecules are verified, with elevated leptin receptor and reduced ligand levels in ossified ligament tissues.^129^ These findings strongly support leptin’s significant contribution to the pathological mechanisms underlying spinal ligament ossification. Modulating the leptin signaling may represent a promising approach to retard the development of OPLL.

### Sex hormones

The relation between estrogen and bone metabolism has been extensively studied, particularly in the context of osteoporosis. However, its role in spinal ligament ossification has received comparatively less attention. Previous clinical observations reveal a distinct pattern of sex hormone dysregulation in male patients with OPLL, marked by elevated total estrogen levels and decreased 5a-(OH)2 testosterone concentrations. The results also show a positive correlation between these hormonal alterations with the degree of ossification progression.^130^ Nevertheless, this phenomenon appears specific to males, as female patients, despite experiencing more dramatic hormonal fluctuations during menstrual cycles and menopause, do not exhibit similar changes.^130^ Experiments have demonstrated that ligament cells from OPLL patients possess estrogen receptors with enhanced binding affinity for estrogen compared to those from non-OPLL individuals.^131^ Genetic analyses further indicate that variations in estrogen receptor genes may influence both OPLL predisposition and disease severity.^132^ Supporting evidence from animal models suggests that sex hormone imbalances can affect the development of chondrocytes and fibroblasts during the integration of the posterior longitudinal ligament with vertebral structures.^133^ Notably, recent research on human periodontal ligament cells provides additional mechanistic insights, showing estrogen’s capacity to promote osteogenic differentiation.^134,135^ Despite these findings, the precise involvement of sex hormones in pathological ossification processes remains unconfirmed, highlighting the need for further investigation.

## Challenges and perspectives

Ossification of the posterior longitudinal ligament represents a complex multifactorial disorder with polygenic inheritance, which may account for the current limitations in elucidating its fundamental pathogenic mechanisms. Despite extensive investigation, research has yet to achieve sufficient depth or convergence to fully characterize the disease’s pathophysiology. Existing epidemiological studies examining the association between endocrine-metabolic factors and OPLL are significantly constrained by several limitations, including inadequate sample sizes, uncontrolled confounding variables, selection bias, geographic and ethnic restrictions, and inconsistent assessment protocols. These methodological shortcomings have resulted in divergent conclusions across studies, ultimately compromising their scientific validity and cross-study comparability. To address these issues, there is an urgent need for standardized, large-scale epidemiological investigations across diverse populations and geographic regions to validate the endocrine-metabolic factors implicated in the onset and progression of OPLL.

Despite significant advancements in OPLL research, the precise molecular mechanisms through which various endocrine-metabolic factors contribute to OPLL development remain incompletely understood, highlighting the necessity for more fundamental mechanistic studies. Emerging evidence indicates that leptin interacts with components of the insulin signaling cascade through recruitment of insulin receptor substrates,^136–138^ suggesting potential crosstalk between leptin and insulin signaling pathways. Clinical observations further support this interaction, as demonstrated by the positive correlation between leptin/BMI ratio and serum insulin levels in female OPLL patients.^122^ These findings suggest that elevated levels of both leptin and insulin may synergistically enhance downstream signaling pathway, thereby contributing to OPLL pathogenesis. Given the frequent co-occurrence of various metabolic disorders such as obesity and diabetes mellitus in OPLL patients, the interaction between leptin and insulin pathways presents a constructive perspective for future research to focus on the synergistic effects of multiple metabolic regulators. Beyond endocrine-metabolic factors, comprehensive understanding of OPLL pathogenesis will require systematic investigation of the interplay between genetic predisposition, metabolic dysregulation, and biomechanical stress factors.

From a clinical perspective, elucidating the precise roles of endocrine-metabolic factors in OPLL development could significantly advance both early intervention strategies and therapeutic approaches. Detailed characterization of metabolic marker dynamics throughout OPLL progression may facilitate the identification of reliable biomarkers for disease monitoring and prognosis prediction. Mechanistic understanding of these factors’ specific roles in OPLL pathogenesis could inform the development of targeted therapies directed against key molecular mediators. Besides, the timely correction of endocrine-metabolic disturbances through restoration of metabolic homeostasis may offer a promising therapeutic strategy for OPLL management.

## Conclusion

This systematic review synthesizes contemporary research from domestic and international sources to critically examine the role of endocrine-metabolic factors in the pathogenesis and progression of OPLL. Substantial progress has been achieved in elucidating the roles of metabolic disorders, such as dysregulated glucose metabolism (e.g., hyperglycemia, insulin resistance, AGEs), lipid metabolism (e.g., dyslipidemia), and bone-mineral homeostasis (e.g., calcium-phosphate disturbance, aberrant bone remodeling) in OPLL development. While the precise molecular mechanisms underlying their contribution to OPLL pathogenesis remain incompletely characterized, current investigations have significantly advanced our understanding of OPLL from a metabolic perspective, providing critical insights into its multifactorial etiology. Continued exploration of these endocrine-metabolic relationships promises to deepen fundamental insights into OPLL pathogenesis while simultaneously informing the development of targeted therapeutic strategies, including metabolic modulation and precision interventions for clinical management.
